# The human auditory system uses amplitude modulation to distinguish music from speech

**DOI:** 10.1371/journal.pbio.3002631

**Published:** 2024-05-28

**Authors:** Andrew Chang, Xiangbin Teng, M. Florencia Assaneo, David Poeppel

**Affiliations:** 1 Department of Psychology, New York University, New York, New York, United States of America; 2 Department of Psychology, Chinese University of Hong Kong, Hong Kong SAR, China; 3 Instituto de Neurobiología, Universidad Nacional Autónoma de México, Juriquilla, Querétaro, México; 4 Ernst Struengmann Institute for Neuroscience, Frankfurt am Main, Germany; 5 Center for Language, Music, and Emotion (CLaME), New York University, New York, New York, United States of America; 6 Music and Audio Research Lab (MARL), New York University, New York, New York, United States of America; Universidad de Salamanca, SPAIN

## Abstract

Music and speech are complex and distinct auditory signals that are both foundational to the human experience. The mechanisms underpinning each domain are widely investigated. However, what perceptual mechanism transforms a sound into music or speech and how *basic* acoustic information is required to distinguish between them remain open questions. Here, we hypothesized that a sound’s amplitude modulation (AM), an essential temporal acoustic feature driving the auditory system across processing levels, is critical for distinguishing music and speech. Specifically, in contrast to paradigms using naturalistic acoustic signals (that can be challenging to interpret), we used a noise-probing approach to untangle the auditory mechanism: If AM rate and regularity are critical for perceptually distinguishing music and speech, judging artificially noise-synthesized ambiguous audio signals should align with their AM parameters. Across 4 experiments (*N* = 335), signals with a higher peak AM frequency tend to be judged as speech, lower as music. Interestingly, this principle is consistently used by all listeners for speech judgments, but only by musically sophisticated listeners for music. In addition, signals with more regular AM are judged as music over speech, and this feature is more critical for music judgment, regardless of musical sophistication. The data suggest that the auditory system can rely on a low-level acoustic property as basic as AM to distinguish music from speech, a simple principle that provokes both neurophysiological and evolutionary experiments and speculations.

## Abstract 2

What enables the human auditory system to distinguish between music and speech? Through a reductionist approach involving judgments on artificially generated ambiguous noise clips, this study shows that the auditory system relies on basic acoustic parameters, such as amplitude modulation rates and regularities, to differentiate between music and speech.

## Introduction

Music and speech, two complex auditory signals, are frequently compared across many levels of biological sciences, ranging from system and cognitive neuroscience to comparative and evolutionary biology. As acoustic signals, they exhibit a range of interesting similarities (e.g., temporal structure [1,2]) and differences (e.g., music, but not speech, features discrete pitch intervals). In the brain, they are processed by both shared [3–6] and specialized [7–10] neural substrates. However, which acoustic information underpins a sound to be perceived as music or speech remains an open question.

One way to address the broader question of how music and speech are organized in the human mind/brain is to capitalize on ecologically valid, “real” signals, a more holistic approach. That strategy has the advantage of working with stimulus materials that are naturalistic and, therefore, engage the perceptual and neural systems in a typical manner (e.g., [11,12]). The disadvantage of adopting such an experimental attack is that it can be quite challenging to identify and isolate the components and processes that underpin perception. Here, we pursue the alternative reductionist approach: parametrically generating and manipulating ambiguous auditory stimuli with basic, analytically tractable amplitude modulation (AM) features. If the auditory system distinguishes music and speech according to the low-level acoustic parameters, the music/speech judgment on artificially noise-synthesized ambiguous audio signals should align with their AM parameters, even if no real music or speech is contained in the signal.

In the neural domain, AM is a basic acoustic feature that drives auditory neuronal circuits and underlying complex communicative functions across both humans and nonhuman animals. At the micro- and meso-levels, single-cell and population recording of auditory cortex neurons in nonhuman animals demonstrated various mechanisms to encode AM features (e.g., [13,14]). At the macro-level, human neuroimaging studies showed that the acoustic AM synchronizes the neural activities at auditory cortex and correlated with perception and speech comprehensions (e.g., [15–18]). A critical but underexplored gap is the mechanism of how low-level AM features affect a sound to be processed as a complex high-level signal such as music and speech.

**Fig 1:**
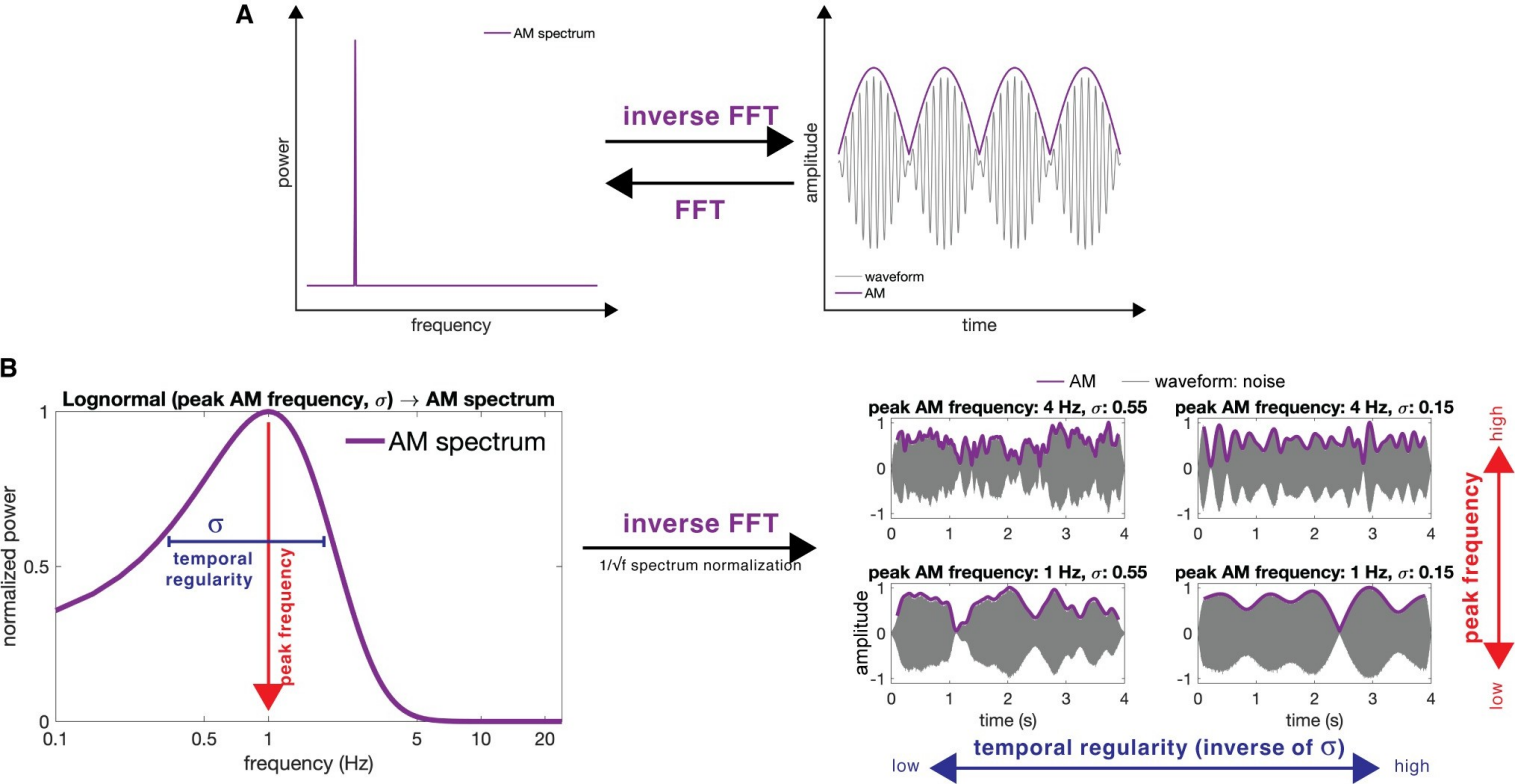
Pipeline for stimulus generation. (**A**) Illustration of amplitude modulation (AM) and AM spectrum. The right panel shows a sound waveform (gray line); the outline of the waveform shows the amplitude envelope or AM (purple line), which conceptually corresponds to loudness fluctuation over time. The fast Fourier transformation (FFT) transforms a time-domain AM signal into a frequency-domain power spectrum (left panel). The inverse FFT transforms a frequency-domain AM spectrum to a time-domain AM signal. (**B**) We use a lognormal function to generate AM spectra with different peak frequency and temporal regularity (σ) parameters (left panel). A smaller σ results in a narrower lognormal function in the spectrum, which means that the time-domain AM signal fluctuates at a more constrained frequency range and is, therefore, more temporally regular. After $1/\sqrt{frequency}$ spectrum normalization, the inverse FFT is applied to transform AM spectra into AM signals. Each AM signal is used to modulate a noise carrier to generate the final stimulus. Each stimulus sounds like white noise with fluctuating amplitude (right panel). Using this pipeline, we generate sound excerpts with parametrically designated AM properties (peak frequency and temporal regularity). See Methods for further details.

Our experiments tested the hypothesis that a remarkably basic acoustic parameter can, *in part*, determine a sound to be perceptually judged as music or speech. The conjecture is that AM (Fig 1) is one crucial acoustic factor to distinguish music and speech. Previous studies that quantified many hours and a wide variety of music and speech recordings showed distinct peak AM rates in the modulation spectrum: music peaks at 1 to 2 Hz and speech peaks at 3.5 to 5.5 Hz [19–21]. Consistent with those findings, these rate differences are also observed in spontaneous speech and music production [22]. Next, temporal regularity of AM could also be important, as music is often metrically organized with an underlying beat, whereas speech is not periodic and is better considered quasirhythmic [20,23]. Also, supporting the relevant role played by AM, neuroimaging evidence showed that temporally scrambled but spectrally intact signals weaken neural activity in speech- or music-related cortical clusters [9,24]. Finally, a preliminary study (*n* = 12) showed that listeners were able to near-perfectly categorize 1-channel noise-vocoded realistic speech and music excerpts [19]. However, the noise-vocoding approach was insufficient to mechanistically pinpoint the degree to which AM rate and regularity contribute to music/speech distinction, as this manipulation preserved all the envelope temporal features above and beyond rate and regularity. For example, onset sharpness of speech envelope is encoded by the spoken language cortical network (superior temporal gyrus) and critical to comprehension [25–27]; also, the onset sharpness of the music envelope is crucial for timbre perception, e.g., a piano tone typically has a sharper onset than violin. We therefore build on the notion that the AM distinction between music and speech signals appears to be acoustically robust. However, in order to advance our understanding of potential mechanisms, we ask what aspects of the AM influence listeners to make this perceptual distinction. How acoustically reduced and simple can a signal be and still be judged to be speech or music?

Based on the literature, we hypothesized that stimuli with a lower-in-modulation-frequency and narrower-in-variance peak (i.e., higher temporal regularity, more isochrony) in the AM spectrum would be judged as music, while those with higher and broader peaks (i.e., lower temporal regularity) as speech. If these hypotheses are plausible, artificial sounds synthesized with the designated AM properties should be perceptually categorized accordingly. This noise-probing approach is conceptually similar to the reverse-correlation approach in studies seeking to understand what features are driving the “black-box” perceptual system (e.g., [28,29]). In short, we synthesized stimuli with specific AM parameters by “reversing” a pipeline for analyzing realistic, naturalistic music and speech recordings (Fig 1B). First, we used a lognormal function that resembles the empirically determined AM spectra reported in previous studies [19,20]; this function permits the independent manipulation of peak frequency and temporal regularity parameters. Next, after transforming each AM spectrum into a time-domain AM signal (inverse Fourier transform), that signal was used to modulate a flat white noise (i.e., low-noise noise) carrier to generate a 4-s duration experimental stimulus. This approach, importantly, eliminates typical spectral features of *both* music and speech. In our 4 online experiments, participants were told that each stimulus came from a real music or speech recording but was synthesized with noise, and their task was to judge whether it was music or speech. Although none of the stimuli sounded like real music or speech, participants’ judgments revealed how well each stimulus matched their internal representation of one or the other perceptual category.

## Results

**Fig 2:**
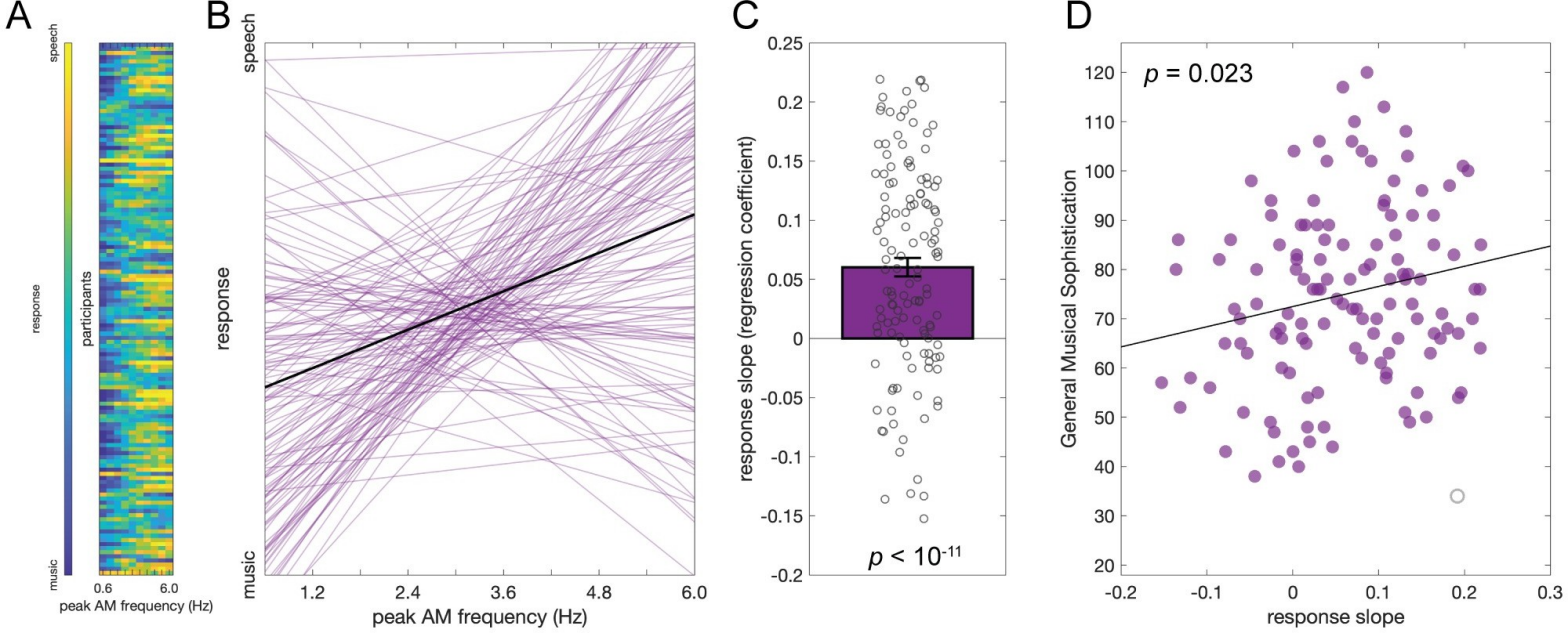
Results of Experiment 1. (**A**) The music vs. speech judgment response of each participant at different levels of AM peak frequencies. (**B**) Fitted regression lines of each participant’s response. (**C**) Each dot represents the response slope on peak frequencies of a participant, and the bar and the error bars represent the mean ± standard error. The participants’ response slopes were significantly above 0, suggesting that the participants tend to judge the stimuli with a higher peak AM frequency as speech and lower as music. (**D**) The response slopes and the General Musical Sophistication score of the participants were positively correlated, suggesting that the musically more sophisticated participants are more likely to judge the stimuli with a higher peak AM frequency as speech and a lower peak frequency as music. Note that the gray circle marks the outlier, and the regression line and the *p*-value reported on the figure were based on the analysis without the outlier. Underlying data and scripts are available at https://doi.org/10.17605/OSF.IO/RDTGC and in S1 Data.

In Experiment 1, we manipulated peak AM frequency while σ (the regularity parameter, or the width of the peak of the AM spectrum; see Methods) was fixed at 0.35 (the value was chosen as it sounded the most “natural” or “comfortable” according to the informal feedback from colleagues in the lab). Stimuli were presented one at a time, and participants were requested to judge whether a stimulus is music or speech. Data from 129 participants were included in the analyses. The overall responses are presented in Fig 2A. To investigate the effect of peak frequencies, each participant’s responses (speech = 1, music = 0) were linearly regressed on the peak frequencies (mean ± standard error of *R*^*2*^ = 0.53 ± 0.03; Fig 2B). The response slopes were significantly above 0 (Fig 2C; *t*(128) = 7.70, *p* < 10^−11^, Cohen’s *d* = 0.68), suggesting that people judge sounds with a higher peak AM frequency as speech and sounds with a lower peak AM frequency as music. We then explored the association of this judgment with participants’ musical sophistication and found that the participants with a higher General Musical Sophistication score (Gold-MSI [30]; see Methods) were more likely to have a higher response slope (*r*(127) = 0.17, *p* = 0.056; but after removing 1 outlier: *r*(126) = 0.20, *p* = 0.023; Fig 2D). We further split the participants by slope at 0 and performed an unequal-variance 2-sample *t* test without removing that 1 outlier. This analysis confirmed that the participants with a positive response slope have higher General Musical Sophistication scores than the participants with a negative slope (*t*(57.25) = 2.96, *p* = 0.005, Cohen’s *d* = 0.57). We further correlated the response slope with each subscale of the musical sophistication index, but none of them were significant (unsigned *r*(127) < 0.16, *p* > 0.075). While null effects should be interpreted with caution, this suggests that general musical sophistication, rather than a specific musical aspect, is driving the outcome. In short, the findings show that the sounds with a higher peak AM frequency are more likely to be judged as speech and lower as music, and this tendency is positively associated with participants’ general musical sophistication.

Note that we attempted to fit the data with a logistic psychometric function. Although the findings were consistent as the fitted slopes of the logistic model were also significantly above 0 (*t*(128) = 6.85, *p* < 10^−9^), suggesting the sounds with a higher peak AM frequency are more likely to be judged as speech over music, the *R*^*2*^ of the logistic model were much lower than the linear model (mean *R*^*2*^ difference: 0.19), so did the following experiments (see Methods for more details), suggesting that the linear model was a more appropriate model. Therefore, only the linear models were interpreted.

**Fig 3:**
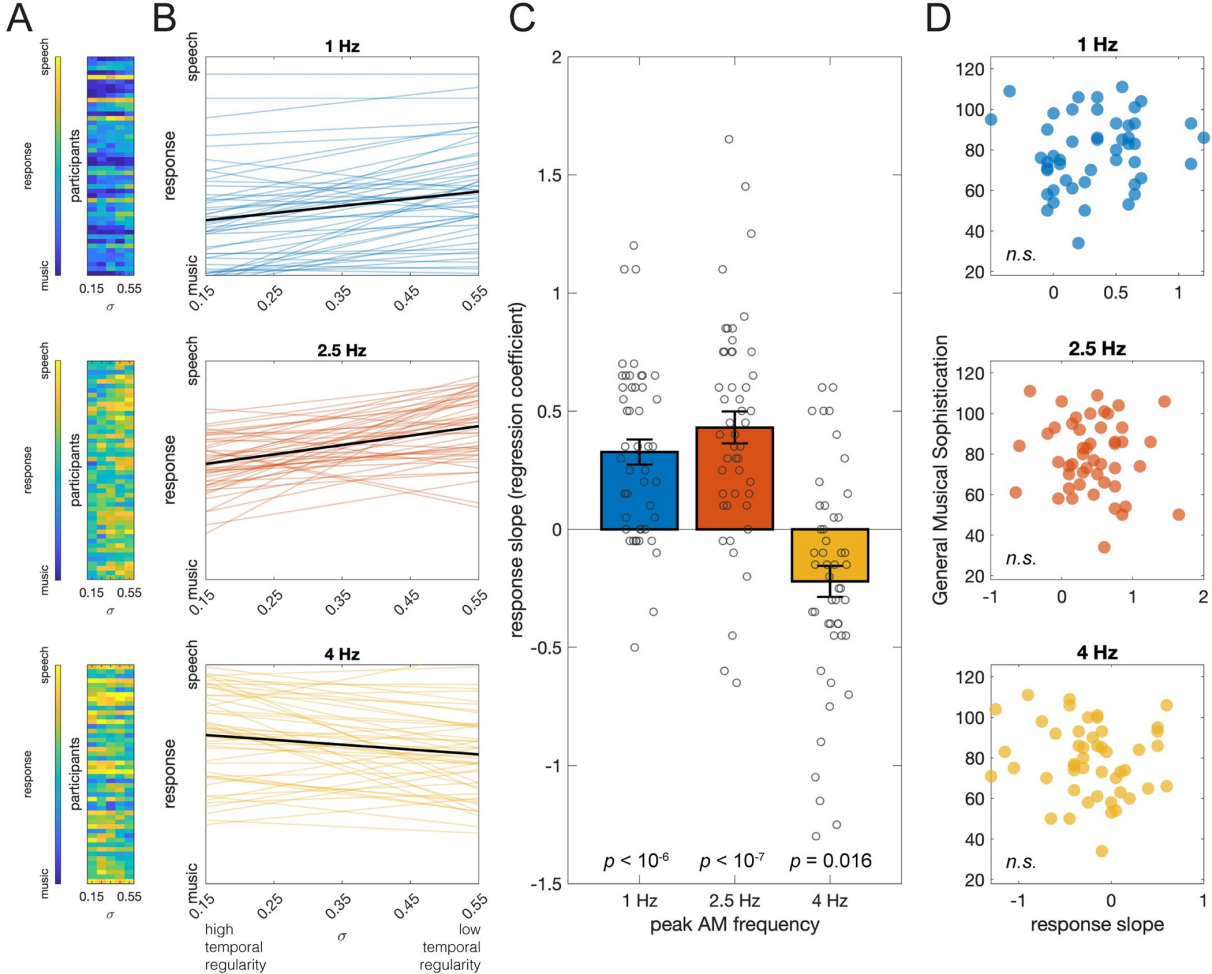
Results of Experiment 2. (**A**) The music vs. speech judgment response of each participant at different levels of temporal regularity (σ). (**B**) Fitted regression lines of each participant’s response. (**C**) The participants’ response slopes on σ were significantly above 0 for the peak AM frequencies at 1 and 2.5 Hz but not 4 Hz. This suggests that participants tend to judge the temporally more regular stimuli as music and irregular as speech, but this tendency was not observed when the peak frequency was as high as 4 Hz. (**D**) The response slopes and the General Musical Sophistication scores were not correlated at any peak AM frequencies. Underlying data and scripts are available at https://doi.org/10.17605/OSF.IO/RDTGC and in S1 Data. *n*.*s*., nonsignificant.

To investigate the effect of temporal regularity, in Experiment 2, we manipulated AM temporal regularity (σ) at 3 peak AM frequencies (1, 2.5, and 4 Hz, which roughly correspond to the AM range of music, a midpoint, and speech). The procedure was identical to Experiment 1, and data from 48 participants were included. The overall responses are presented in Fig 3A. Each participant’s responses were linearly regressed on the σ under each peak frequency (*R*^*2*^ = 0.37 ± 0.02; Fig 3B). The response slopes were significantly above 0 for the peak frequency at 1 Hz (*t*(47) = 6.19, *p* < 10^−6^, Cohen’s *d* = 0.89) and 2.5 Hz (*t*(47) = 6.37, *p* < 10^−7^, Cohen’s *d* = 0.92), suggesting that listeners tend to judge sounds with lower temporal regularity (higher σ) as speech and higher regularity as music (Fig 3C). Note that this pattern was the opposite for the peak frequency at 4 Hz, with a lower effect size (*t*(47) = −3.34, *p* = 0.016, Cohen’s *d* = 0.48). It suggests that the association between temporal regularity and the music judgment is conditional on the low-to-mid peak AM frequency range, and the influence of temporal regularity is weaker when peak AM frequency is in the AM range of speech. We also examined the associations between participants’ musical sophistication levels and response slope, but no correlation was significant (Fig 3D; unsigned *r*(46) < 0.13, *p* = 0.404).

The dichotomy of the behavioral judgment that our task imposes could be a concern because it only allows a stimulus to be judged as music or speech, while ignoring other possible categories. It is, to be sure, reasonable to directly contrast music and speech, as these are arguably among the most dominant high-level auditory forms in human cognition, sharing many commonalities (cf., [1,9]), and a discrimination task between two categories is usually considered psychophysically more powerful than two separate detection tasks on each category [31]. However, other auditory categories, such as animal calls and environmental sounds, are critical in human perception as well. Therefore, we tested the robustness of the findings of Experiments 1 and 2 by replicating them with detection tasks, and we investigated whether there were effects specific to music or speech.

**Fig 4:**
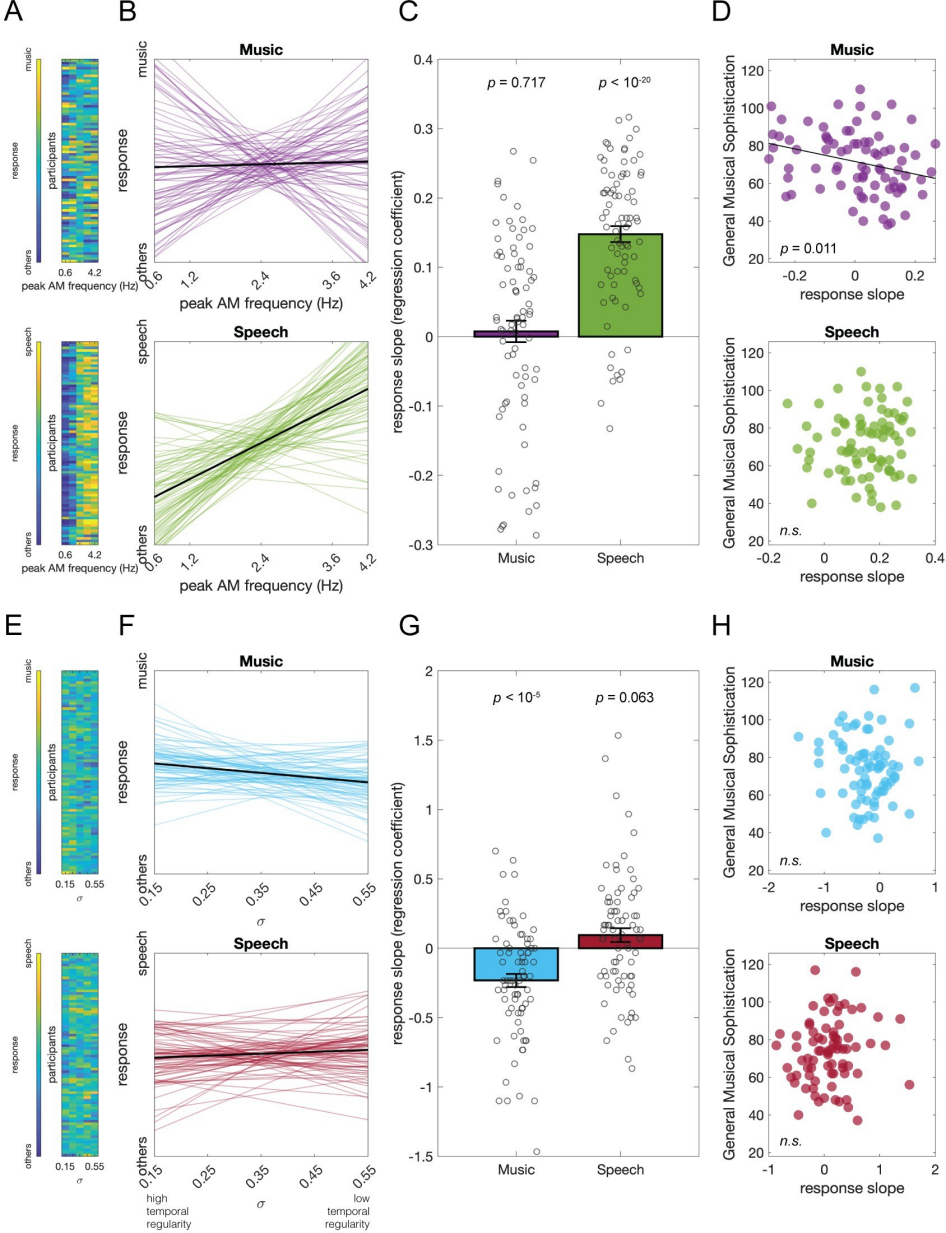
**Results of Experiments 3** (A-D) and **4** (E-H). (**A**) The “music vs. others” and “speech vs. others” judgment response of each participant at different levels of peak AM frequencies. (**B**) The fitted regression line of each participant’s response. (**C**) The participants’ response slopes on peak frequencies were significantly above 0 for the speech task but not for the music task, suggesting that the participants tend to judge the stimuli with a higher peak AM frequency as speech. (**D**) The response slopes and the General Musical Sophistication scores of the participants were positively correlated for the music task but not for the speech task, suggesting that the musically more sophisticated participants are more likely to judge the stimuli with a lower peak AM frequency as music. (**E**-**H**) The same format as above, but at different levels of temporal regularity (σ). The participants tend to judge the stimuli with a higher temporal regularity as music. Underlying data and scripts are available at https://doi.org/10.17605/OSF.IO/RDTGC and in S1 Data. *n*.*s*., nonsignificant.

In Experiment 3, peak AM frequency was manipulated with σ fixed at 0.35; 80 participants were included in the analyses. In the “music detection” task, participants were instructed that 50% of the stimuli were music and 50% were not music (“others”), and they were asked to judge whether it was music or something else. For the “speech detection” task, the task was analogous. The 50% instruction was added to prevent participants with a strong response bias. Each participant performed both tasks with the same stimuli. The overall responses are presented in Fig 4A. Each participant’s responses (music or speech = 1, others = 0) were linearly regressed on peak frequency for each task (*R*^*2*^ = 0.68 ± 0.02; Fig 4B). For the speech task, the response slopes were significantly above 0 (*t*(79) = 12.79, *p* < 10^−20^, Cohen’s *d* = 1.43; Fig 4C), suggesting that the sounds with a higher peak AM frequencies are more likely to be judged as speech over others. Musical sophistication did not correlate with the speech response slope (*r*(78) = 0.04, *p* = 0.717; Fig 4D). For the music task, the response slope was not significantly different from 0 (*t*(79) = 0.49, *p* = 0.628, Cohen’s *d* = 0.05; Fig 4C). Interestingly, there was a significant correlation suggesting that the more musically sophisticated participants are more likely to judge the sound with a lower peak AM frequency as music (*r*(78) = −0.28, *p* = 0.011; Fig 4D), and this is again confirmed by the unequal-variance 2-sample *t* test between split-data at slope equals to 0 (*t*(72.57) = 2.66, *p* = 0.010, Cohen’s *d* = 0.58). We also correlated the response slope with each subscale; however, once again, none of them passed the Bonferroni-corrected statistical threshold at 0.01 (unsigned *r*(78) < 0.28, *p* > 0.013). Together, the effect of peak AM frequency reported in Experiment 1 is robustly replicated for the speech judgment, but the music judgment was conditional on participants’ general musical sophistication.

In Experiment 4, AM temporal regularity was manipulated while the peak AM frequency was fixed at 2 Hz (equally likely to be judged as music or speech, according to the previous experiments). The tasks were as in Experiment 3, and data from 78 participants were included. The overall responses are shown in Fig 4E. Each participant’s responses were linearly regressed on σ for each task (*R*^*2*^ = 0.32 ± 0.02; Fig 4F). For the music task, the response slope was significantly below 0 (*t*(77) = -4.95, *p* < 10^−5^, Cohen’s *d* = 0.56; Fig 4G), suggesting that people tend to judge the sounds with higher temporal regularity (lower σ) as music. For the speech task, the response slopes were slightly above 0 but not reaching the statistical threshold (*t*(77) = 1.89, *p* = 0.063, Cohen’s *d* = 0.21; Fig 4G). We did not observe any associations between participants’ musical sophistication and response slope (unsigned *r*(76) < 0.13, *p* > 0.293; Fig 4H). Together, the effect of AM temporal regularity reported in Experiment 2 was robustly replicated for music but only a trend was observed for speech.

## Discussion

These surprising findings and their replications show that listeners use acoustic amplitude modulations in sounds, one of the most basic features, fundamental to human auditory perception, to judge whether a sound is “like” music or speech, even when spectral features are eliminated. We show that peak AM frequency can affect high-level categorization: Sounds with a higher peak AM frequency tend to be judged as speech, those with a lower peak as music, especially among musically sophisticated participants. This pattern is consistent with previous quantifications of natural music recordings showing that the peak AM frequency of music is lower than speech [19]. This result might arise because of participants’ (implicit) knowledge of this acoustic feature. We note that, while the effect of peak AM frequency in Experiment 1 was robustly replicated in the speech task in Experiment 3, in the music task, the effect was more salient among musically sophisticated participants but not visible when pooling all participants. In other words, peak AM frequency is a universal cue for speech but not for music. A possible explanation is that this effect depends on listeners’ experience or sophistication with music or speech sounds. While our participants exhibited a ceiling effect for speech (as university students, every listener can be classified as an “expert” in speech), their musical sophistication scores appeared lower than the norm (Experiment 3 versus Müllensiefen and colleagues [30]: 71.39 versus 81.58, Cohen’s *d* = 0.49; but it is similar to other studies (e.g., [32,33])). The potential effect of speech expertise would need to be examined, for example, in future developmental studies in which expertise can be more carefully controlled.

Temporal regularity (and, in the extreme, isochrony, if σ = 0) of AM also has an effect: Sounds with more regular modulation are more likely to be judged as music than speech. This is consistent with the fact that Western music is usually metrically organized while speech is quasirhythmic [20,23]. There are a few aspects worth discussing. First, this effect is more relevant to music than to speech. The detection tasks in Experiment 4 show that the effect of temporal regularity is only robustly observed for music but not for speech. It appears that temporal regularity is a more prevalent principle than peak AM frequency to judge a sound as music as this effect does not depend on the listener’s musical sophistication. Second, in Experiment 2, the effect of temporal regularity was slightly opposite when the peak AM frequency was at 4 Hz. A possible explanation is that temporal regularity might be less critical for distinguishing music and speech when peak AM frequency is already in the canonical speech range 3.5 to 5.5 Hz [19–21]. Last but not least, while temporal regularity in the current parameter range did not drastically influence the auditory judgments, the current data demonstrate a clear pattern across participants: A sound with a more temporally regular AM is more like music.

AM is one of the most fundamental building blocks for auditory perception, and especially so for human speech. While frequency/spectral information is critical for auditory object identification, pitch perception, and timbre, AM is considered a key information-bearing component and critical for speech intelligibility [34,35]. AM, especially around the 2-4 Hz, is faithfully encoded by neurons in the primary auditory cortex [14,36]. While previous studies have demonstrated that temporal envelope information alone is arguably sufficient for speech perception (e.g., [37]), the current findings further show that AM rate can be used to identify a sound as speech or not (i.e., Fig 4C). Relatedly, AM rate helps identify music, at least among musically sophisticated listeners. This could be for different reasons. First, music has salient features in both time and frequency domains. A recent survey showed that adults explicitly consider both AM regularity (rhythm/beat) and melody (frequency/spectral domain), but not AM rate, as being the primary acoustic features for distinguishing speech and song [38]. This is consistent with the current finding that people rely on AM regularity more than rate to identify music. Second, the association between AM rate and music perception might require musical experience. This is consistent with the neural entrainment studies showing that the fidelity of auditory cortex entraining to music rhythm is positively associated with the musical expertise of the listeners [25,39]. Together, our data provide the empirical advance that AM rate or regularity alone, regardless of the fine temporal features (e.g., onset sharpness) preserved by the noise-vocoded approach [19], have an effect on the music/speech judgment. Given that the AM rate and regularity are processed early in the auditory cortex [14], notably prior to superior temporal gyrus encoding of speech onset (e.g., [26,27]), AM rate or regularity should have more decisive roles than temporal envelope features for distinguishing music and speech at an early stage of the auditory cortical pathway.

The current study has four noteworthy limitations. First, the lognormal function can resemble the average AM spectrum of many hours of music or speech recordings [19], but it does not necessarily approximate *individual* recordings well. Second, the current forced-choice task design can only demonstrate how acoustic features affect the auditory *judgments*, but whether participants subjectively experienced the *percepts* of our stimuli as “reduced” forms of music or speech is unclear, as the rich spectral and timbral features of typical music or speech were by design eliminated from the stimuli. Third, while the current experimental design only showed the influences of AM rate and regularity on distinguishing music and speech, we did not compare their influences to those of other acoustic features. Although spectral or frequency modulation, orthogonal to AM, is another promising acoustic feature fundamental to auditory perception, the current study focuses on only the AM aspect as it has been demonstrated distinct between music and speech acoustics while the spectral aspect has not. Lastly, the factors that contributed to the substantial individual differences in music-related tasks remain unclear, and musical sophistication only partially accounts for it. Other perceptual and cognitive factors (e.g., preference for fast or slow music, unawareness of hearing loss among young adults) and experimental factors (e.g., whether the participants were exposed to any specific music or speech in the environment while performing our experiment online, remotely, and on their own) likely contributed to the individual differences as well. Nevertheless, our reductionist approach demonstrates the striking fact that music or speech judgment starts from basic acoustic features such as AM.

A related phenomenon that builds on the role of temporal structure can be illuminated by these data. The speech-to-song illusion demonstrates that, by looping a (real) speech excerpt, the perceptual judgment can gradually shift from speech toward song [40–42]. The effects reported here are consistent with the speech-to-song illusion: The low frequency power of the AM spectrum would emerge from the repeating-segment periodicity and, therefore, bias the judgment toward music. Supporting this view, this illusion disappears if speech is temporally jumbled in every repetition [40], which eliminates low-frequency periodicity across repetitions. Furthermore, consistent with our findings, the strength of the illusion is also positively associated with beat regularity and participants’ musical expertise [41,43–45].

The properties of AM that support the distinction of music and speech merit consideration in the context of human evolution and neurophysiology. Group cohesion and interpersonal interaction have been hypothesized as one primary function of music [46–53]. If music serves as an auditory cue for coordinating group behaviors, predictable temporal regularity at the optimal rate for human movements and audiomotor synchronization (1 to 2 Hz; [54–56]) would be important. And, in fact, motor brain networks are involved while processing auditory rhythms (e.g., [57–64]). The AM rate of speech, analogously, has been attributed to the neurophysiological properties of the specialized auditory-motor oscillatory network for speech perception and production, as well as the associated biomechanics of the articulatory movements [17,20,65,66]. Consistent with these data patterns, perceptual studies have also shown a general pattern that music versus speech task performance is optimal with rates ranging around 0.5 to 6.7 and 2 to 9 Hz, respectively [67,68].

The experimental results we present demonstrate that human listeners can use a basic acoustic feature fundamental to auditory perception to judge whether a sound is like music or speech. These data reveal a potential processing principle that invites both neurophysiological and evolutionary experiments and speculations that could further address the long-lasting questions on the comparison between music and speech in both the humanities and the sciences.

## Methods

### Resource availability

All stimuli, experimental programs, raw data, and analysis codes have been deposited at a publicly available OSF repository (https://doi.org/10.17605/OSF.IO/RDTGC).

### Participants

The participants were students at New York University who signed up for the studies via the SONA online platform and received course credit for completing the experiments. The local Institutional Review Board (New York University’s Committee on Activities Involving Human Subjects) approved all protocols (IRB-FY2016-1357), in complete adherence to the principles outlined in the Declaration of Helsinki. All participants provided informed consent via an online form. Participants had self-reported normal hearing, were at least 18 years old, and reported no cognitive, developmental, neurological, psychiatric, or speech-language disorders. The total number of online participants was 488, and the data of 335 participants (208 females, 122 males, 5 other/prefer not to say, age range: 18 to 25) were included for analysis (see Quantification and statistical analysis for exclusion criteria, and Results for the sample size of each experiment).

### Stimuli

The pipeline to generate audio stimuli with a designated peak AM frequency and temporal regularity parameters is composed of the following steps (resembling an inverse pipeline for analyzing audio recordings), which are conceptually illustrated in Fig 1.

1. A lognormal function, $Lognormal(x;\mu ,\sigma^{2},b)=\frac{1}{(x-b)\sigma \sqrt{2\pi }}exp\left(-\frac{{\left(\ln(x-b\right)-\mu )}^{2}}{2\sigma^{2}}\right)$, was used to generate signals that are similar to typical, averaged AM spectra of music and speech recordings (based on the data in [19]). *x* is the spectrum frequency. The relation between the parameter *μ* and the peak frequency (mode or *m*) is $\mu =\ln\left(m-b\right)+\sigma^{2}$. The parameter σ is the standard deviation of the function’s natural logarithm. A smaller *σ* represents a spectrum with power more narrowly concentrated around a peak, which will result in higher temporal regularity of the signal, as there is only one dominant frequency. The parameter *b* is an x-scale shift parameter as a function of m: *b = −1*.2813*m, which enables nonzero intercepts to better approximate the AM spectra reported in a previous study [19].
2. The lognormal function was multiplied by $1/\sqrt{x}$ (reversing the operation in [19]) to build an AM spectrum.
3. An inverse fast Fourier transformation with random phases was applied to an AM spectrum to generate a 20-s time-domain signal with a 44.1-kHz sampling rate, and then it was transformed to an amplitude envelope [69,70].
4. The resulting amplitude envelope was used to modulate a 20- to 20,000-Hz low-noise noise (LNN) carrier sound. The LNN is a white noise with a flat amplitude envelope [71,72], which ensures that the amplitude fluctuations of the final stimuli were not caused by the carrier signal.
5. The middle 4-s segment of each 20-s amplitude-modulated LNN was extracted as a stimulus.
6. There were 100, 50, 50, and 50 stimuli generated for each condition of Experiments 1 to 4, respectively, and the root-mean-square values of all the stimuli were equalized within each experiment. All steps were performed using MATLAB R2020a.

### Procedure

The experiments were programmed on PsychoPy Builder (v2020.1.2) and executed on the Pavlovia.org platform.

The participants were required to perform the experiment using a browser on their personal computer, in a quiet environment with headphones on, and each listener could set the audio volume at a comfortable level. First, only those participants who passed a headphone screening task (see below) could proceed. Next, the practice phase included 4 trials; the AM parameters of these stimuli were within the range of, but not identical to, the parameter values used in the subsequent testing phase. On each practice trial, a stimulus was presented, and then participants were asked to make a binary judgment by clicking a button on the screen, without time limit. After the response, the next trial started. A probe trial was inserted in the practice phase, which presented 1 to 4 brief tones without warning in a 2-s window with random stimulus-onset asynchronies, and the participants were requested to indicate the number of tones by pressing the corresponding key. Participants could repeat the practice phase until they felt comfortable to proceed to the testing phase. Only in Experiments 3 and 4, a practice phase was inserted prior to each of the first music and speech blocks.

In the testing phase, for Experiments 1 and 2, for each participant, a set of 150 unique stimuli (15 or 10 per condition in Experiments 1 or 2, respectively) were randomly drawn from the stimulus pool, and they were randomly ordered within each of the first and second half of the experiment, resulting in a total of 300 testing trials. There was no cue between two halves of the experiment. The participants were not instructed regarding the occurrence rates of “music” or “speech.”

For Experiments 3 and 4, within each of the first and second halves of the experiment, there were 1 music block and 1 speech block, randomly ordered. Within each block, there were 75 unique stimuli (15 per condition) randomly drawn from the stimulus pool, and the same set of stimuli was used for all 4 blocks for each participant, resulting in a total of 150 trials for each task and, therefore, totaling 300 testing trials for the entire experiment. Before and during each block, there were text and visual cues on the screen to remind the participants of the current block type. The participants were instructed that 50% of the trials were music or speech and 50% were not music or speech (“others”), respectively, for each block type.

For all the experiments, the procedure of each testing trial was identical to the practice trial. A self-paced break was inserted every 10 trials, and the percentage of progress in the experiment was shown on the screen during the break. Twelve probe trials were mixed with roughly even spaces with the testing trials.

After the experiment, participants were directed to another webpage to anonymously fill out demographic information, the Goldsmiths musical sophistication index, and other background and task-related questions (not analyzed).

#### Headphone screening task

The participants were requested to perform a headphone screening task prior to the main task, to ensure that they used headphones to complete our online experiments [73]. On each trial, participants were asked to identify the quietest tone (3-alternative forced choice) among three 1-s duration 200 Hz pure tones (with 100 ms ramps), including a binaurally in-phase loud tone, an antiphase loud tone, and an in-phase quiet tone of (−6 dB). Stimuli were presented sequentially with counterbalanced orders across 6 trials. Because the antiphase loud tone would be attenuated by phase cancelation in the air if it was played through loudspeakers, the quietest tone can only be correctly identified with headphones. Participants had to perform at least 5 out of 6 trials correctly to proceed.

#### Goldsmith musical sophistication index (Gold-MSI)

The Gold-MSI is one of the most common and reliable indices and for assessing musicality [30]. It is composed of 39 questions to assess multiple aspects of music expertise, including active engagement, perceptual abilities, musical training, singing abilities, and emotional responses. The General Musical Sophistication subscale is a general index that covers all the aspects of Gold-MSI, which ranges from 18 to 126; the mean and the standard deviation of the norm (147,633 participants) are 81.58 and 20.62, and the reliability α is 0.926.

### Quantification and statistical analysis

Since all participants completed the study online without supervision, we used several exclusion criteria to ensure data quality. (1) The participants who did not complete both the experiment *and* the questionnaire, who did not pass the headphone screening task, admitted not using headphones throughout the experiment, made the same response for all the trials, or whose probe trial accuracy below 90%, were excluded. These criteria excluded 41, 19, 31, and 23 participants from Experiments 1, 2, 3, and 4. (2) Since the participants were instructed that the occurrence rate of music/speech was 50% in Experiments 3 and 4, the participants whose response biases exceeded 50 ± 15% in any task were excluded. This criterion excluded 16 and 23 participants from Experiments 3 and 4. Statistical test significance was assessed with α = .05, two-tailed. The specific tests used are reported in the Results section. The computations were performed on MATLAB R2020a and R2021b.

A logistic psychometric model f(x;a,b)=1/(1+exp(−b*(x−a))) was attempted to fit to the response data of each participant, while the parameter *a* was bounded between the extreme stimuli levels of each experiment (for example, 0.6 and 6.0 Hz for Experiment 1). However, the fitted *R*^*2*^ values of logistic model were lower than the *R*^*2*^ of the linear model across all experiments (mean *R*^*2*^ difference ≥ 0.19), suggesting that linear model is a better choice than logistic model to fit the current dataset.

### Power analysis and sample sizes

As the effect size of this task was unknown, in the Experiment 1, we recruited more than 100 participants to reduce the risk of being underpowered and to estimate the statistical power for the following experiments. Based on the data of Experiment 1, a power analysis showed that the required number of participants was 20 when alpha level was set at 0.05 and statistical power at 0.8, and 36 when alpha level was set at 0.01 and statistical power at 0.9. Therefore, we targeted the sample size of Experiment 2 to be slightly above those levels (*n* > 40). Although the tasks of Experiments 3 and 4 were similar to Experiments 1 and 2, the judgment of “speech versus others” and “music versus others” might have a lower statistical power than “music versus speech,” as “others” is not a well-defined category. Therefore, we set the target sample sizes to be double (*n* ≈ 80) as the required sample size of alpha at 0.01 and power at 0.9.

## Supporting information

S1 Data Data underlying the plots in Fig 2–4. (XLSX)
